# Cooperative Stabilization of Pickering Emulsions by Starch and Chitin Nanoparticles: Roles of Ball-Milling, Gelatinization, Adsorption, and Viscosity Behavior

**DOI:** 10.1021/acsomega.5c11090

**Published:** 2026-02-11

**Authors:** Matheus de Oliveira Barros, Carolina Siqueira Franco Picone, Yi Lu, Edy Sousa de Brito, Morsyleide de Freitas Rosa, Orlando J. Rojas

**Affiliations:** † Federal University of Ceará (UFC), Department of Chemical Engineering, 60355-636 Fortaleza, Ceara, Brazil; ‡ Universidade Estadual de Campinas (UNICAMP), Department of Food Engineering and Technology, 13083-970 Campinas, São Paulo, Brazil; § Bioproducts Institute, 8166 The University of British Columbia, 2385 East Mall, Vancouver, BC V6T 1Z4, Canada; ∥ Department of Chemical and Biological Engineering, University of British Columbia, 2360 East Mall, Vancouver, BC V6T 1Z3, Canada; ⊥ Embrapa Food and Territories, Rua Cincinato Pinto 348, Maceió 57020-050, Alagoas, Brazil; # Embrapa Tropical Agroindustry, Rua Dra Sara Mesquita 2270, 60020-181 Fortaleza, Ceara, Brazil; ¶ Department of Chemistry, University of British Columbia, 2036 Main Mall, Vancouver, BC V6T 1Z1, Canada; ∇ Department of Wood Science, University of British Columbia, 2424 Main Mall, Vancouver, BC V6T 1Z4, Canada

## Abstract

We demonstrate enhanced Pickering emulsion stabilization by modified starch nanoparticles (SNP) through their combination with chitin nanocrystals (ChNC). The effect of the biopolymer’s ratio on the emulsion stabilization mechanisms was elucidated based on their role at the interface and bulk phases. The stabilization achieved with a 1:1 (SNP:ChNC) ratio surpasses that of other ratios studied, exhibiting a synergistic effect compared with neat SNP and the 10:1 and 5:1 systems. Emulsion stability was further improved by applying thermal pretreatment to the aqueous phase before homogenization. The heat-treated 1:1 emulsion maintained a consistent droplet size (∼3.4 μm) over four months, even after slow creaming. This stability is attributed to SNP adsorption at the oil–water interface, providing a mechanical barrier to droplet’s coalescence, while ChNC is also present in the oil–water interface and forms a colloidal network in the continuous phase, hindering droplet’s mobility and preventing Ostwald ripening. These findings expand the application of chitin as an emulsifier and address the rising demand for healthier and sustainable emulsified formulations with reduced costs.

## Graphical abstract

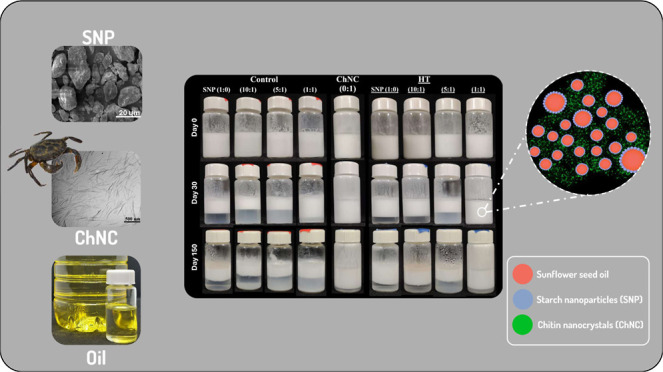

## 1 Introduction

Emulsions, consisting of a liquid continuous phase with dispersed droplets, are inherently thermodynamically unstable. To achieve kinetic stability, emulsions benefit from physicochemical and rheological effects. Typically, stability is enhanced by the adsorption of surfactants at the droplet interface.^1–3^

However, traditional petrochemical surfactants present challenges, such as toxicity, foam formation, and interactions with biological systems. Pickering emulsions offer promising alternatives to address these issues. Unlike conventional emulsions, Pickering emulsions are stabilized by solid particles that adsorb at the colloidal interface, providing steric hindrance to coalescence. During homogenization, these particles adsorb to droplet surfaces, acting as physical barriers that prevent coalescence and enhance kinetic stability.^4–7^

Effective Pickering emulsion stabilization requires particles with balanced wettability. Biobased alternatives such as starch have gained increasing interest due to their sustainability and functionality.^8,9^ Starch, a common plant-derived polysaccharide, has a unique semicrystalline structure composed of amylose and amylopectin. Both are glucose polymers linked by glycosidic bonds, yet they differ significantly in structure, properties, and functionality.^10^ Amylose is primarily linear, with glucose units joined by a-1,4 glycosidic bonds and occasional α-1,6 linkages, allowing it to adopt a helical conformation in aqueous solutions stabilized by hydrogen bonding. In contrast, amylopectin is highly branched, with glucose units linked by a-1,4 bonds in linear regions and a-1,6 bonds at branching points. This branching increases the water solubility and molecular weight. While amylose forms firm gels upon cooling, amylopectin imparts a softer, more viscous texture.^11,12^

Recently, starch nanoparticles (SNPs) have been explored for film-forming applications and Pickering emulsion stabilization due to their high surface area and biocompatibility.^13–17^ Smaller starch particles typically provide greater interfacial coverage and denser particle packing at the oil–water interface when present at sufficient concentration, owing to their higher specific surface area.^18–20^ Although the attachment energy of individual particles increases with particle radius, the overall stability of Pickering emulsions reflects a balance between particle adsorption energy, interfacial coverage, packing density, and network formation in the continuous phase.^21^

Hydrolysis is a widely used method for SNP production, but physical approaches, such as ball-milling, are emerging as environmentally favorable alternatives. Ball-milling disrupts the crystalline structure of starch granules, reducing particle size and significantly lowering the temperature, sometimes to ambient levels, of gelatinization, a process that disrupts the double-helix structure of amylose in starch, enhancing its flexibility and dispersibility. This mechanical treatment also decreases starch hydrophilicity, making ball-milling a promising, sustainable technique for SNP production over traditional chemical processes.^14,22–25^

During gelatinization, starch loses its structural integrity, leading to the self-assembly of SNPs into smaller spheres, which allows for a denser packing at the oil–water interface. This structural rearrangement contributes to the improved emulsion stability. Additionally, the presence of free gelatinized starch in the aqueous phase increases the viscosity, offering an additional mechanism for stabilization. Overall, gelatinization plays a crucial role in modifying starch properties to enhance the stability and performance of colloidal systems.^25,26^

To improve the interfacial properties of starch particles, they are often modified with 2-octen-1-ylsuccinic anhydride (OSA), introducing hydrophobic alkyl chains that render the particles amphiphilic. While OSA modification is FDA-approved and considered safe for food applications, its disposal and handling are more challenging compared to acid- or base-treated alternatives.^18,27,28^ Consequently, most food-grade starch-based Pickering systems rely on chemical modification to achieve a sufficient interfacial activity. An optional approach to chemical modification is the combination of biobased particles, leveraging their individual properties to enhance Pickering emulsion stability.

Given the growing demand for sustainable and food-safe formulations, combining naturally derived particles has emerged as a promising alternative to chemical surface modification. Unlike previous strategies based primarily on chemically modified starch or on chitin nanocrystals as the sole stabilizing phase, starch nanoparticles (SNPs) can be coupled with other biobased polymers to compensate for their limited interfacial activity and enhance the robustness of the particle network formed at the oil/water interface. Such hybrid systems exploit complementary physicochemical properties, including differences in surface charge, morphology, and wettability, to achieve superior stabilization without relying on synthetic agents. Among the various biobased candidates, chitin has gained significant attention due to its structural versatility and environmental compatibility.^29–31^

Chitin is a naturally occurring polysaccharide widely distributed in the exoskeletons of arthropods, in the cell walls of fungi, and in certain algae. Structurally, it is composed of β-(1→4)-linked *N*-acetyl-d-glucosamine units, forming a linear polymer with high crystallinity and mechanical strength. Due to its abundance and its biocompatible, biodegradable, and nontoxic nature, chitin has attracted considerable interest as a renewable biomaterial.^32–34^ Recent studies have explored the use of chitin nanocrystals (ChNCs), the rod-like crystalline domains obtained from the partial deacetylation and acid hydrolysis of chitin, as efficient stabilizers in Pickering emulsions. Their nanoscale dimensions, high surface area, and amphiphilic nature enable them to strongly adsorb at the oil–water interface, forming a rigid particle network that enhances emulsion stability.^35–37^

Herein, we propose a system combining ball-milled SNPs and chitin nanocrystals (ChNCs) to stabilize food grade emulsions. In this approach, ChNC compensates for the limited interfacial activity of SNPs, which arises from the partial hydrophobicity imparted during milling, thereby facilitating efficient adsorption at the oil–water interface. Beyond interfacial adsorption, ChNCs contribute to electrostatic stabilization and form a steric barrier that mitigates droplet coalescence through repulsive interactions. Importantly, the incorporation of SNPs enables a reduction in ChNC content, lowering formulation cost and viscosity while offering opportunities to tailor digestion-related properties, as starch is more easily digested than chitin. Thermal treatment of starch may further modulate particle interactions and contribute to the overall stability of the system.^18,19,25^

Despite the extensive literature on Pickering emulsions stabilized by polysaccharides, systematic studies combining nonchemically modified starch nanoparticles (SNPs) with chitin nanocrystals (ChNCs) remain limited. Few works have examined such systems from an application-oriented perspective, where reduced reliance on chitin nanocrystals may potentially contribute to lower formulation costs and where the distinct digestibility profiles of SNPs and ChNCs may offer opportunities for future nutritional tailoring. This work explores synergistic stabilization via adsorption and phase structuring in the absence of surface modification, aligning with the need for safer, sustainable formulations. Our goal is to develop a safe, high-quality formulation suitable for food applications.

## 2 Materials and Methods

### 2.1 Materials

Commercially available corn starch (Kimimo), sunflower seed oil (Mazola) (same batch was used for all experiments), Nile red dye (Sigma-Aldrich), Nile blue (Sigma-Aldrich), fluorescein 5(6)-isothiocyanate (FITC) (Sigma-Aldrich), hydrochloric acid 37% (Sigma-Aldrich), and glacial acetic acid (Sigma-Aldrich) were all used as received.

Commercial corn starch has an approximate molecular weight of 2 × 10^7^ g/mol, which varies with the amylose-to-amylopectin ratio; higher amylopectin increases the molecular weight. The starch used in this study contained 23.16% amylose.^14^

The starch nanoparticles (SNPs) were obtained through ball-milling following our previously published method.^14^ The starch was milled for 20 h using a ceramic system (20 cm diameter) equipped with ceramic balls (1.5 cm) and operated at a starch/ball mass ratio of 1:20 at 21 °C; these conditions were used as they were the best results in size obtained from the previous work. The SNPs as well as commercial starch were fully characterized in a previous publication.^14^

The chitin nanocrystals (ChNC) were obtained following the method proposed by Larbi (2018)^38^ and obtained from chitin extracted from crab shells (*Metacarcinus magister*) according to our previous work.^39^ Briefly, 1 g (dry weight) of ChNC was added to 30 mL of HCl 3 M solution and heated for two hours at 90 °C. The reaction was stopped by dilution with 800 mL of cold Milli-Q water. After centrifuging the ChNC suspension for 15 min at 10,000 rpm and discarding the supernatant, the particles were dialyzed to pH 4. The ChNC suspension (pH 4) was sonicated at 40% intensity for 15 min (5 s on and 2 s off). Finally, the pH was adjusted to 3 with acetic acid, and the suspension was stored until use.

The deacetylation degree of the ChNC was measured to be 22% using standard methodology described by Bai et al.^40^ The degree of acetylation (DA) of chitin and partially deacetylated chitin was determined by FTIR spectroscopy. The DA was calculated from the ratio of the absorbance bands at 1655 cm^–1^ (amide I) and 3450 cm^–1^ (O–H stretching), according to eq 1.

1 $$DA\left(\%\right)=\frac{A_{1655}}{A_{3450}}\times 100$$

Both SNP and ChNC were observed using FEI Tecnai G2 Twin 200 kV TEM, to see their morphology, and their sizes were measured using the GIMP software; the size of the particles was measured using pixel-to-length calibration (version 2.10.36).

### 2.2 Preparation of the Pickering Emulsions

The aqueous phase of the emulsions (1 wt % total solids) was prepared using different SNP:ChNC mass ratios (10:1, 5:1, and 1:1), corresponding, respectively, to decreasing SNP content and increasing ChNC content, with the 1:1 formulation representing the highest ChNC fraction (50 wt % of total particles). Two neat (reference) aqueous phases containing only SNP or ChNC were also prepared. All aqueous phases were adjusted to pH 3 using acetic acid prior to the addition of the oil phase.

To create a 10:90 Pickering emulsion, 10 wt % sunflower oil was added to the aqueous phase; sunflower oil was used in this study because it is a well-known and widely used food-grade oil for emulsion preparation.^41,42^ The mixture was immediately emulsified^43^ using a high shear force mixer (IKA Ultra-Turrax T25 easy clean, Breisgau, Germany) at 12,000 rpm for 1 min. Following homogenization, the emulsions underwent four rounds of 2 min (30 s intervals) sonication (60% potency) in an ice bath to prevent heating of the sample (Sonifier 550, Branson, Connecticut, USA). The emulsions were vortexed for 30 s between each sonication.

Emulsions both with and without a heating treatment of the aqueous phase were studied. For the heat-treated (HT) emulsions, the starch suspension was heated to 60 °C for 10 min to ensure gelatinization of the starch particles before the addition of ChNC, when present, and oil. The non-heat-treated emulsions are referred to as control in this work. In our study, the gelatinization process occurred at room temperature in contrast to typical native starch gelatinization which is carried out at 80 °C. The heat treatment (60 °C), previously mentioned, was applied to ensure that all the particles present in the samples were fully gelatinized.^14^

### 2.3 Kinetic Stability of Emulsions

Emulsion phase separation was evaluated at 21 °C, over five months. The volume of separated phases was measured using GIMP (version 2.10.36) and the separation index (SI) was calculated according to eq 1:

2 $$Separation\;index\left[\%\right]=\left(\frac{H_{S}}{H_{T}}\right)\cdot 100$$

where HT is the total height of the emulsion column and HS is the height of the serum bottom phase.

A LUMiSizer (LUM GmbH, Berlin, Germany) dispersion analyzer was used to determine the creaming velocity and instability index of the emulsion samples and the sedimentation velocity of the aqueous phases. About 430 μL of the sample was placed within the low-volume polypropylene LUMiSizer cuvette. The analysis consisted of 300 10 s runs at 4000 rpm and 21 °C, using an 870 nm wavelength.

### 2.4 Emulsion Morphology

An Eclipse LV100N POL (Nikon, Tokyo, Japan) optical microscope equipped with a camera attachment was used to capture the images of the emulsion droplets as soon as they were prepared. To view the droplets under a bright field, 50 μL of the emulsion sample was placed in a glass slide with a glass cover.

The dyed emulsion was examined using Confocal Laser Scanning Microscopy (Olympus FV1000, Tokyo, Japan) to see how the starch and chitin particles were organized in the system, right after being prepared. Nile Blue (Sigma-Aldrich) was used to dye the SNP, and FITC was used to dye the chitin nanocrystals, in accordance with Lopes et al. (2019) with some adjustments. 100 mL of dehydrated ethanol and 50 mL of FITC solution (0.5 mg of FITC/1 mL of ethanol) were combined with 40 mL of 1 wt % % ChNC suspension (pH 3). The combination was then stirred for three hours in the dark. Following a pH adjustment with NaOH (0.1 M) to 7.0, the solution was centrifuged for 3 min at 5000 rpm and the precipitate was repeatedly washed with Milli-Q water until the dye was no longer visible in the supernatant. The labeled ChNC was freeze-dried and used for emulsion preparation according to Section 2.3.

### 2.5 Droplet Size and Electrostatic Charges

A Mastersizer 3000 (Malvern, Worcestershire, United Kingdom) was used to determine the emulsions’ droplet size distribution. The measurements were performed with the assumption that the droplets were completely spherical. The results were referred to as surface area mean diameter, also known as Sauter mean (D­[3,2]). These measurements were carried out as soon as the emulsions were produced and every 30 days over a 5 month period.

A Zetasizer nano series Nano-ZS (Malvern, Worcestershire, United Kingdom) was used to evaluate the zeta potential (ZP) of samples. To perform the measurements, the emulsions were diluted using Milli-Q water 1:100 with a pH 3 acetic acid solution at 21 °C. Additionally, the ZP of the aqueous phases alone was determined.

### 2.6 Stability Analysis (pH and Temperature)

For the pH stability analysis, 0.5 mL of the HT emulsions were added to 1 mL of a solution with a known pH (3, 5, and 7), with the pH of these solutions being adjusted using HCl. The droplet size and separation index were evaluated immediately after the dilution was prepared and again after 7 days.

For the temperature stability analysis, the emulsions were transferred to 2 mL vials and stored at a constant temperature (4 and 21 °C) for 7 days. The droplet size and creaming index were measured immediately after the emulsions were prepared and once more after 7 days of storage at constant temperatures.

### 2.7 Rheological Properties

The rheological properties of the emulsions were measured using an Anton Paar Modular Compact Rheometer (MCR 302) equipped with a 50 mm diameter parallel plate geometry (PP50) and a 1 mm gap. Flow curve measurements were performed in three steps: (1) a shear rate ramp from 0.1 to 300 s^–1^, (2) a return to 0.1 s^–1^, and (3) a second ramp from 0.1 to 300 s^–1^. All measurements were conducted at 21 °C.

The power-law model (eq 2) was fitted to the last shear rate ramp via least-squares regression. The apparent viscosity (η_ap_) was calculated at 100 s^–1^ using eq 3.

3 $$\tau =k\cdot {\mathrm{\gamma ̇}}^{n}$$

4 $$\eta_{ap}=k\cdot {\mathrm{\gamma ̇}}^{n-1}$$

where τ is the shear stress (Pa), γ̇ is the shear rate (s^–1^), *k* is the consistency index (Pa.s), and *n* is the behavior index (dimensionless).

The time-dependent rheological behavior was assessed by calculating the sample’s hysteresis loop area (Δ*A*), obtained from the integrated areas under the first and second shear ramps, as shown in eq 4:

5 $$\Delta A=\frac{\left(A_{1st}-A_{2nd}\right)}{A_{2nd}}$$

where *A*1st and *A*2nd are the integrated areas under the first and second shear ramps, respectively. The resulting Δ*A* value represents the hysteresis loop area, which is commonly used to distinguish between thixotropic and rheopectic behaviors.

### 2.8 Statistical Treatment

All experiments were conducted with a minimum of three replicates and analyzed using one-way ANOVA followed by Tukey’s posthoc test, performed in OriginLab Pro 2025, to determine statistically significant differences between group means (p-value <0.05).

## 3 Results and Discussion

### 3.1 SNP and ChNC Morphology and Size

Figure 1 shows TEM images of the SNP and ChNC particles. The SNP particles displayed a size of 119 ± 32 nm. As also observed by Liu et al.^44^ and Xu et al.,^25^ the gelatinized starch tends to self-assemble in spherical shapes (Figure 1A). However, a key difference with previous works is the fact that SNPs did not undergo thermal treatment but gelatinized at room temperature.^14^

**Figure 1:**
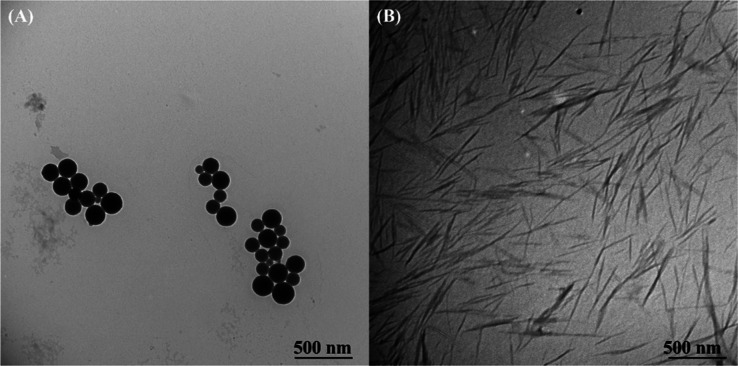
Transmission electron microscopy (TEM) images of starch nanoparticles (A) and chitin nanocrystals (B).

The ChNCs have a length of 252 ± 50 nm and a width of 22 ± 4 nm (spectral ratio of 12 ± 3). The small size of the particles endows high specific surface area and structuring that contributes to the stabilization of the oil–water interface in colloidal systems.^3,28,38,39,45^

### 3.2 Zeta Potential

Both “Control” and “HT” treatments were considered, namely, emulsions in which the aqueous phase did not go through heating and emulsions that used a heat-treated aqueous phase, respectively.

Overall, the zeta potential of the emulsions (Figure 2A) and their respective aqueous phases (Figure 2B) increased as the SNP:ChNC ratio rose (1:1 being the biggest and 1:0 being the smallest). This stems from chitin’s highly positive charge at pH 3.0, due to its protonated amino groups.^36^ As ChNC proportion increases, its contribution to the overall charge increases, raising the zeta potential, seeing as the SNP zeta is close to neutral charge.

**Figure 2:**
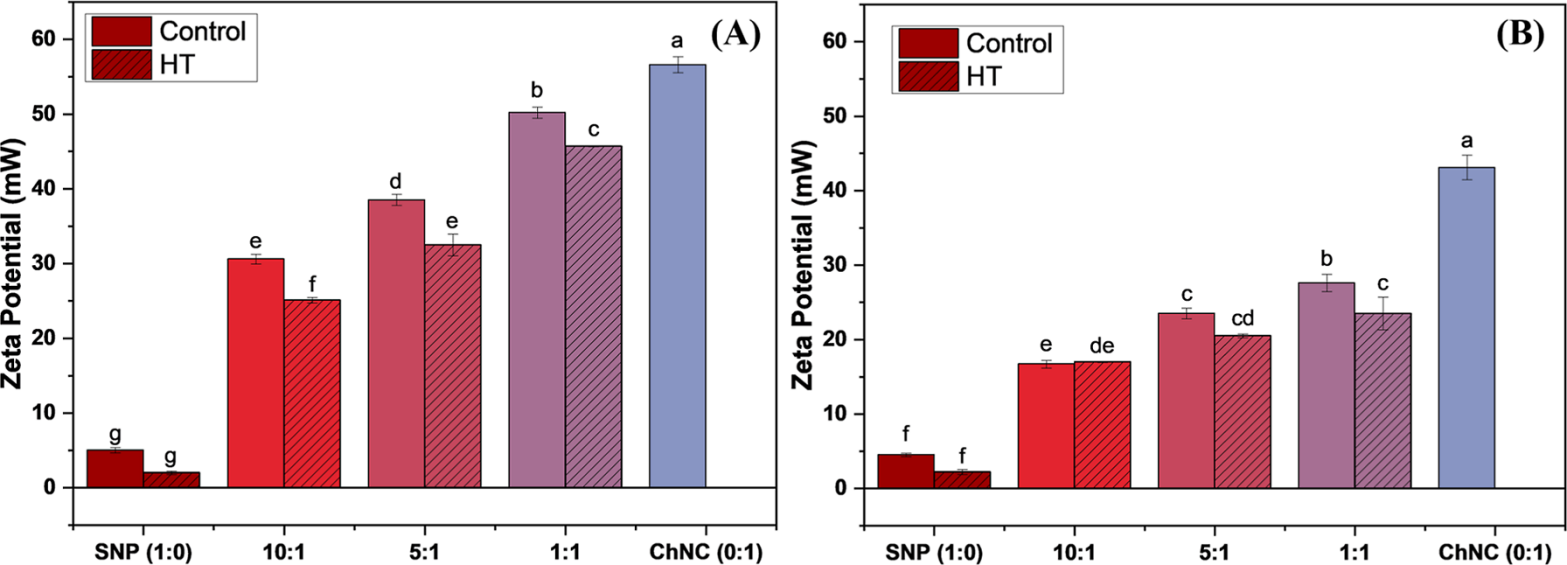
Zeta potential measurements of the different SNP:ChNC ratio emulsions (A) and their respective aqueous phases (B), for control and HT samples (in hatched). Bars with different letters within the same graph are statistically different at *p* < 0.05.

The HT samples exhibited an overall lower zeta potential than the control samples, a phenomenon due to the swelling of the starch granule and the partial breakage of the double-helix starch structure. This process likely resulted in a more flexible and smaller particle with exposed hydroxyl groups that can interact with the positive charge of the chitin, decreasing the zeta potential.^25^ Unlike particles such as cellulose nanocrystals, which typically carry a strong negative surface charge, starch nanoparticles (SNPs) are essentially neutral, whereas chitin nanocrystals (ChNC) exhibit a strongly positive surface charge. This charge asymmetry is insufficient to promote strong electrostatic binding or the formation of a new composite particle. Instead, SNPs and ChNC act alongside one another in the stabilization process, with the limited electrostatic interaction allowing SNPs to rearrange freely and adopt optimal positions within the system, as observed in the microscopy images (Figure 4).

**Figure 3:**
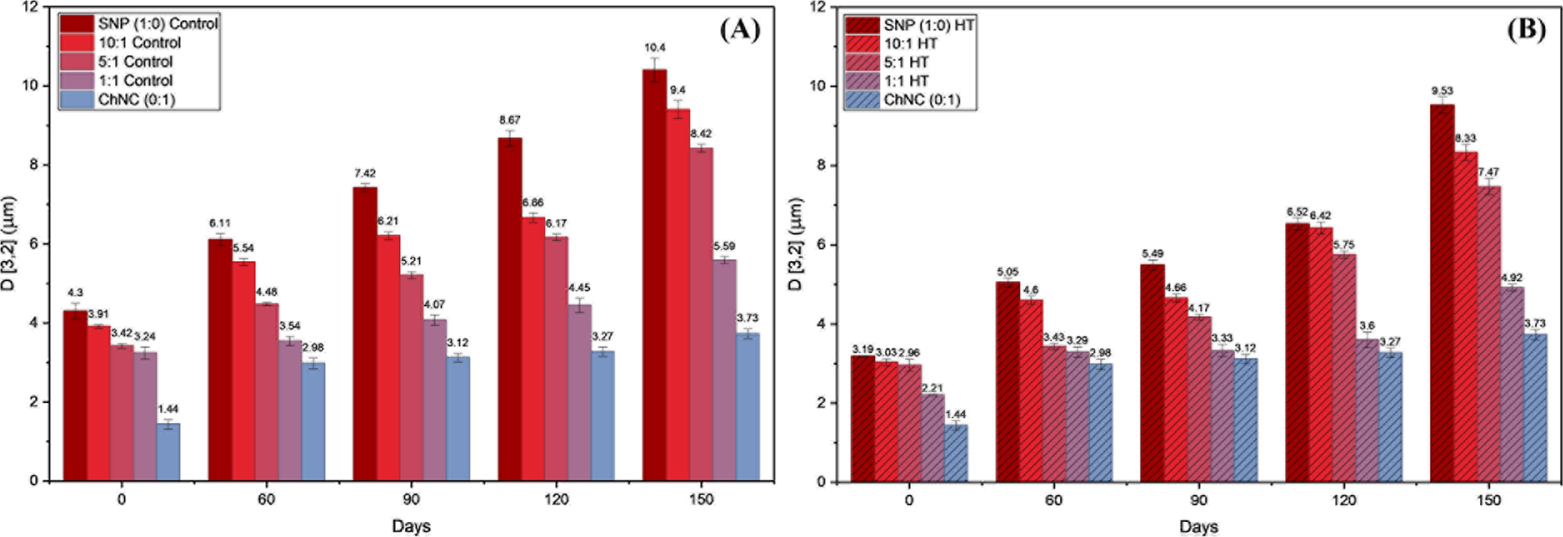
Droplet size based on area of droplets for the different SNP:ChNC ratio emulsions for 150 days storage at 21 °C, for control (A) and HT (B) samples.

**Figure 4:**
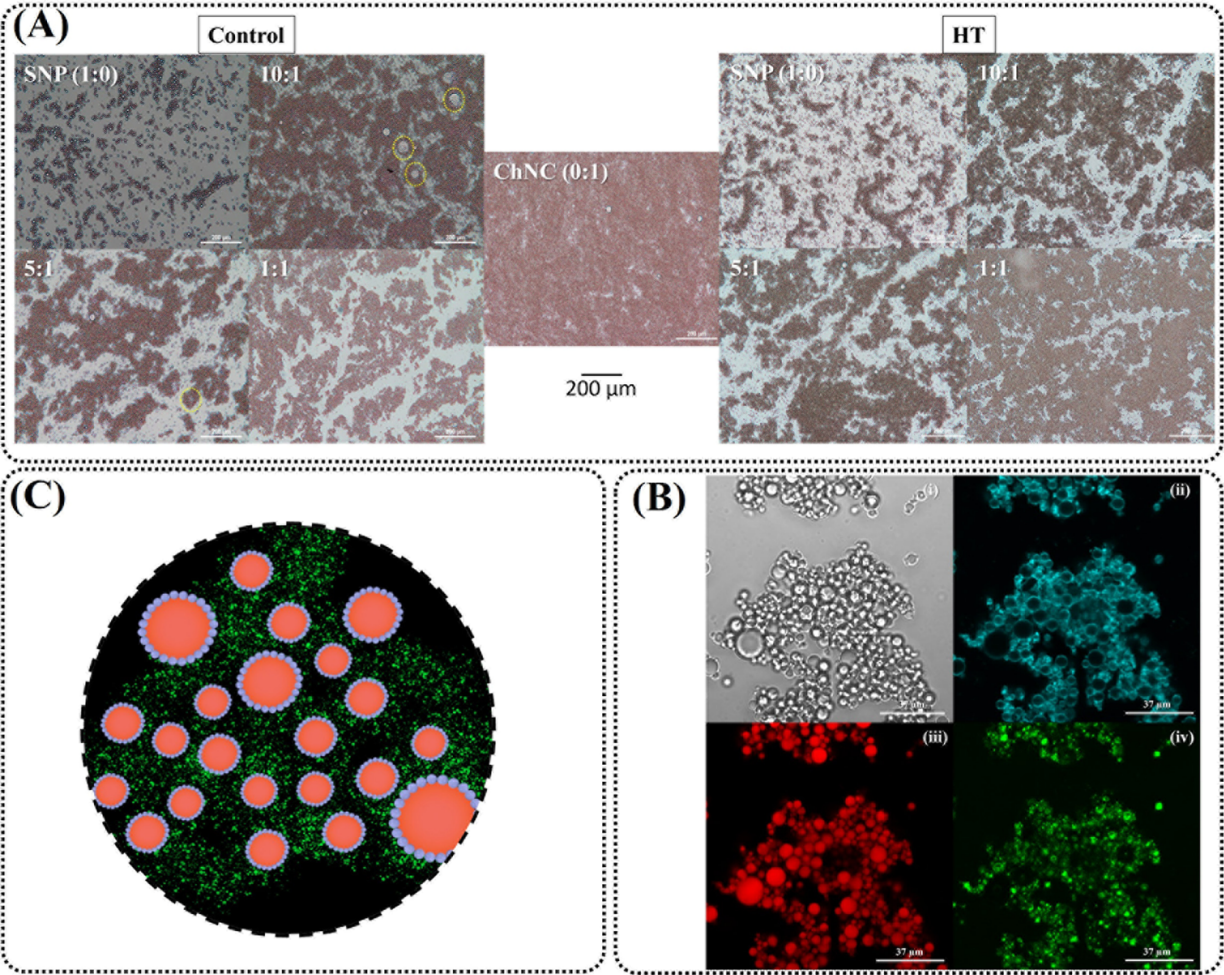
(A) Optical microscopy of the different SNP:ChNC ratio emulsions, both control and HT, taken at day 0. Dashed line shows the presence of large starch granules not engaged in emulsion stabilization. (B) Confocal Laser Scanning Microscopy (CLSM) images of the 1:1 HT sample, at the same day of emulsification; (i) bright-field image of the emulsion; (ii) SNP particles stained with Nile Blue; (iii) sunflower oil droplets in red; (iv) ChNC in green stained with FITC. (C) Schematic representation of the illustrative and conceptual guide of the positioning of SNP (blue) and ChNC (green) around the oil droplets (red) dispersed in water (black).

The same behavior is observed in the aqueous phase of the emulsions (Figure 2B), which displayed a higher zeta potential at higher ChNC fractions and a smaller zeta potential for the HT samples. In general, emulsions exhibit lower zeta potential than the aqueous phases, likely due to the anionic nature of the oil–water interface. Most food oils (such as soybean, sunflower, and olive oil) exhibit a negatively charged interface, due to the hydroxyl groups of fatty acids projecting into the aqueous phase. Thus, these negatively charged groups interact electrostatically with the protonated groups of chitins, thereby reducing the zeta potential of the emulsion relative to the aqueous phase.^46^

These electrostatic conditions suggest that the partial attraction between positively charged ChNC and the hydroxyl-rich SNP surface may enhance the network formation in the aqueous phase, especially in the 1:1 HT formulation. Such interactions reinforce the observed structural synergy between the two biopolymers.

### 3.3 Droplet Size Measurements

The emulsions studied had droplet sizes (droplet volume-area diameter, D 3,2) ranging from 1.4 to 4.3 μm on the day of preparation (Figures 3 and 4). The droplet sizes observed fall within the expected range for Pickering emulsions and are on the smaller side compared to other studies, which report droplet sizes as large as 40 μm for Pickering emulsions stabilized by starch nanocrystals.^19,47^

For the heat-treated (HT) samples (Figure 3B), droplet sizes were consistently smaller than those of the nontreated control samples (Figure 3A) throughout the evaluated period, highlighting the strong impact of thermal treatment on emulsion formation and stability. In particular, the 1:1 HT formulation exhibited droplet sizes comparable to those of the ChNC-only system and maintained high stability over time (Section 3.7). The heat-treated SNP tends to self-assemble into micelles which easily attach to the droplet surface.^44^ These micelles are more flexible and smaller than the initial SNP which can lead to better coverage of smaller oil droplets. This behavior is responsible for the overall smaller droplet sizes on the HT samples as also observed in Figure 1A, showing that a simple step such as a heat treatment yields very good results in droplet increase prevention and emulsion stability. The SNP stabilization paired with the interdroplet stability provided by the ChNC grants the emulsions good overtime stability.

There is a clear and well-known relationship between increased stability and smaller emulsion droplets. Coalescence is responsible for the gradual increase in the droplet size in emulsions over time. An instability process in the system is triggered when these larger, merging droplets rise to the top of the emulsion due to their increased buoyancy.^3,6,48^

As anticipated, all samples’ droplet sizes increased following the 150 days of room temperature storage (Figure 3), leading to emulsion creaming (Figure 8). However, none of the sample emulsions experienced formation of a pure oil phase before 90 days of storage, despite the increased droplet size. Again, the 1:1 HT sample deserves special attention since, after 150 days of storage, its droplet size increased by 122%, a much lesser rise than that of the ChNC-only emulsion, which exhibited a 159% increase. The addition of SNP enables a reduction in ChNC content while maintaining comparable stability, lowering formulation cost, and allowing the potential modulation of properties such as viscosity and digestion behavior, which are properties important for food applications. The emulsion’s droplet size remained within the range of typical Pickering emulsions even after 150 days of storage.

### 3.4 Emulsion Droplet Morphology

The optical microscopy for ChNC-only emulsion, HT, and control emulsions is displayed in Figure 4A. Some intact starch granules (indicated by yellow dashed lines) were observed in the 10:1 and 5:1 control samples, which appeared to have a limited contribution to emulsion stabilization. It was possible to see nearly whole starch granules in the control samples’ microscopy, particularly, which are not engaged in emulsion stabilization. In a previously published study, we observed that the starch is broken up by ball-milling, leaving a polydisperse particle size, which can account for the presence of these large starch granules.^14^

The heat treatment of starch guarantees its complete gelatinization and results in smaller and more flexible starch particles that tend to self-assemble into spherical shapes. This thermal process addresses the issue of large starch granules (Figure 4A) and enhances the emulsion stability. Consequently, no large starch granules were observed in the images of the heat-treated (HT) samples. The samples with an intermediate concentration of chitin nanocrystals (ChNC) exhibited increased flocculation, which can be attributed to their lower zeta potential (Figure 2) and decreased electrostatic repulsion between the droplets.

Confocal microscopy (CLSM) was used in the best formulation containing both SNP and ChNC to better understand the disposition of the particles in the sample that had the most promising stability results among the emulsions made with SNP and ChNC, the 1:1 HT sample (Figure 4B). An illustrative and conceptual schematic representation of the disposition of these particles is shown in Figure 4C to serve as a visual guide, alongside the CLSM images, where both particles are present.

SNPs (blue) are more present in the interface of the oil droplet (red), granting the emulsion a physical barrier against coalescence on an interface level (Figure 4B,C). Conversely, the ChNC (green) is more present in the space between droplets, forming a network of whiskers, adding to the overall stability of the system via lowering the mobility of the droplets, which in turn leads to less coalescence as well [Figure 4B­(iv)]. Both biobased polymers act in the stabilization of the emulsions but through two completely different stabilization mechanisms, which further supports the hypothesis that both these polymers have a synergistic relationship when used combined in Pickering emulsions stabilization.^39,49^

Overall, the emulsions with ChNC have a more compact droplet disposition. The 1:1 HT sample’s densely packed droplet disposition can be attributed to ChNC’s propensity to form bridges between the oil droplets, adding an additional layer of steric stability to the system, as seen in Figure 4B.^49,50^

### 3.5 Rheological Properties

The higher viscosities seen in the 1:1 HT and ChNC-only aqueous phases suggest that aqueous phase thickening plays a significant role in interdroplet stabilization. This viscosity enhancement is not only attributed to the gel-like network formed by ChNC, especially at acidic pH (<6.8), but also complemented by conformational rearrangements in amylose and amylopectin chains due to gelatinization of starch.^51,52^ These molecular changes contribute to an overall increase in the viscosity of HT systems, which, in turn, reinforces emulsion stability.

Apparent viscosity values (Table 1) for heat-treated (HT) emulsions were, in most cases, slightly higher than their nontreated counterparts, an expected result due to complete gelatinization of starch granules promoted by the thermal treatment. This enhancement is attributed to the strong thickening ability of gelatinized starch (SNP), which, when combined with ChNC, results in a more entangled and cohesive aqueous network.^25^

**Table 1:** Viscosity (η or η_ap_), Consistency Index (*k*), Behavior Index (*n*), *R* ^2^ Values, and Flow Behavior of the Studied Emulsions, Both Heat-Treated and Not Heat-Treated^a^

sample	*η* or *η*_ap_ (mPa.s)	*k* (mPa)	*n* (−)	flow behavior	*R* ^2^ (−)
**SNP** (1:0)	2.55 ± 0.04^d^	–	–	Newtonian	0.9951 ± 0.0002^bc^
**10:1**	6.05 ± 0.03^c^	30.79 ± 0.22^c^	0.647 ± 0.002^a^	power law	0.9975 ± 0.0010^ab^
**5:1**	6.82 ± 0.10^c^	37.63 ± 0.31^c^	0.629 ± 0.003^b^	power law	0.9966 ± 0.0017^abc^
**1:1**	9.15 ± 0.03^b^	57.10 ± 0.35^b^	0.602 ± 0.001^c^	power law	0.9985 ± 0.0003^a^
**SNP** (1:0)**HT**	3.10 ± 0.04^d^	–	–	Newtonian	0.9939 ± 0.0013^c^
10:1**HT**	6.30 ± 0.16^c^	33.37 ± 0.27^c^	0.638 ± 0.004a^b^	power law	0.9982 ± 0.0005^ab^
5:1**HT**	7.03 ± 0.03^c^	37.94 ± 0.85^c^	0.634 ± 0.005a^b^	power law	0.9983 ± 0.0004^ab^
1:1**HT**	9.75 ± 0.04^b^	67.34 ± 0.36^b^	0.580 ± 0.001^d^	power law	0.9986 ± 0.0002^a^
**ChNC** (0:1)	2.48 ± 0.09^a^	51.74 ± 0.88^a^	0.340 ± 0.006^e^	power law	0.9962 ± 0.0014^abc^

a Results with different letters in the same row indicate statistically significant differences (*p* < 0.05).

The ChNC emulsion displayed the highest viscosity among all the samples; this is due to the gel forming effect that ChNC has when used in the stabilization of Pickering emulsions specially in lower pH values (<6,8).^36,53^ The higher viscosity values (1:1 HT and ChNC) can be directly associated with the bridge-forming behavior of the ChNC when applied in colloidal systems. The values of viscosity obtained for the emulsions studied were within the range reported in the literature for starch-stabilized Pickering emulsions. The low viscosity of the samples is of good interest for functional beverages applications.^54^

Beyond viscosity, the area of hysteresis analyses provided additional insights into the microstructural stability and flow behavior of the emulsions (Figure 5). Although shear recovery tests and oscillatory rheology were not conducted for all samples, hysteresis loop analysis already captures the extent of structure breakdown and rebuilding under shear, allowing qualitative assessment of thixotropic or rheopectic behavior.

**Figure 5:**
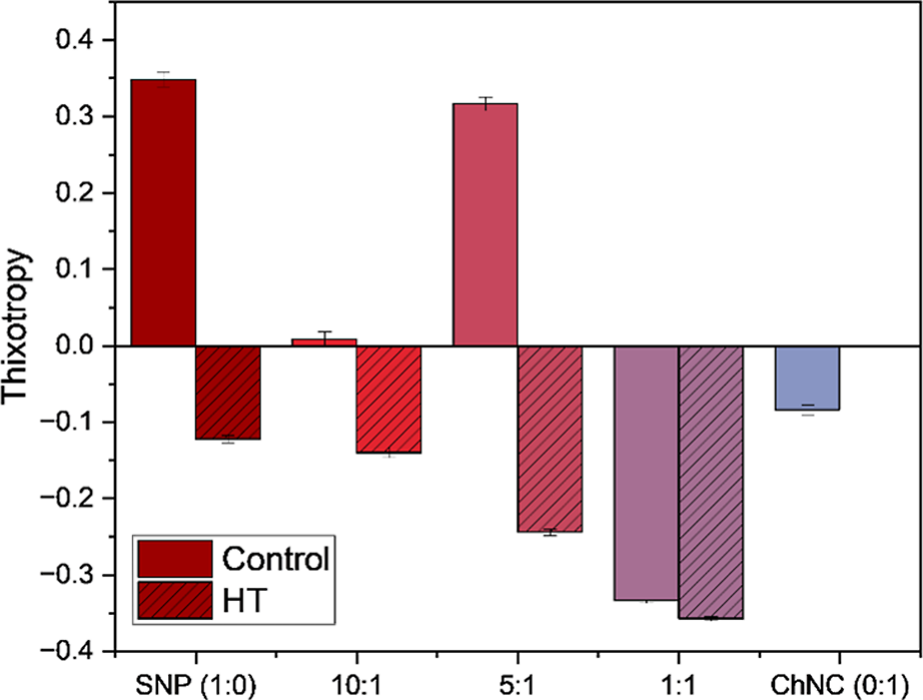
Area of hysteresis measurements for the different SNP:ChNC ratios for both control and HT samples.

The area of hysteresis (dimensionless) of ChNC emulsions is dependent on its hierarchical structure.^55^ While 1:0 SNP control emulsions (starch-only with no heat treatment) exhibited thixotropic behavior (Δ*A* ≈+0.35), indicating a weak structure that breaks under shear, those with balanced or higher ChNC content, especially 1:1 and HT variants, showed rheopectic behavior (e.g., Δ*A* = −0.36 in 1:1 HT). This rheopectic behavior suggests shear-induced structuring, where the system develops more order under flow rather than collapsing. The interplay between high yield stress and negative thixotropy supports the view that these emulsions possess a reversible, resilient internal structure that reorganizes rather than disintegrates during shear, particularly in heat treated, ChNC-rich formulations.^56–58^ Future work incorporating small-amplitude oscillatory shear, yield stress determination, and shear recovery protocols will allow a more quantitative description of this behavior.

### 3.6 Instability Index, Creaming Velocity, and Sedimentation

Centrifugal separation analysis, which the LUMiSzer uses to record the variation of transmitted light over time and space, provides information about the kinetic separation process of the analyzed samples. Results like the instability index, creaming velocity, and sedimentation velocity can be obtained; these parameters are excellent for comparing the kinetic stability between samples.^59^

The instability index of the ChNC-only emulsion was the lowest one compared to all other samples (Figure 6A). The addition of chitin initially has an adverse effect on the instability index of the HT emulsions which corroborate with the visual results of stability over time (Figure 8), but when the concentration of SNP and ChNC was the same, sample 1:1, the instability index was lower for both control and HT samples. Between the SNP and ChNC samples, the 1:1 sample exhibited lower instability that indicates higher emulsion stability. As these measurements were performed in accelerating conditions, the properties of the bulk phase are of fundamental relevance; thus, the role of ChNC and SNP on increasing the viscosity of the aqueous phase is evidenced.

**Figure 6:**
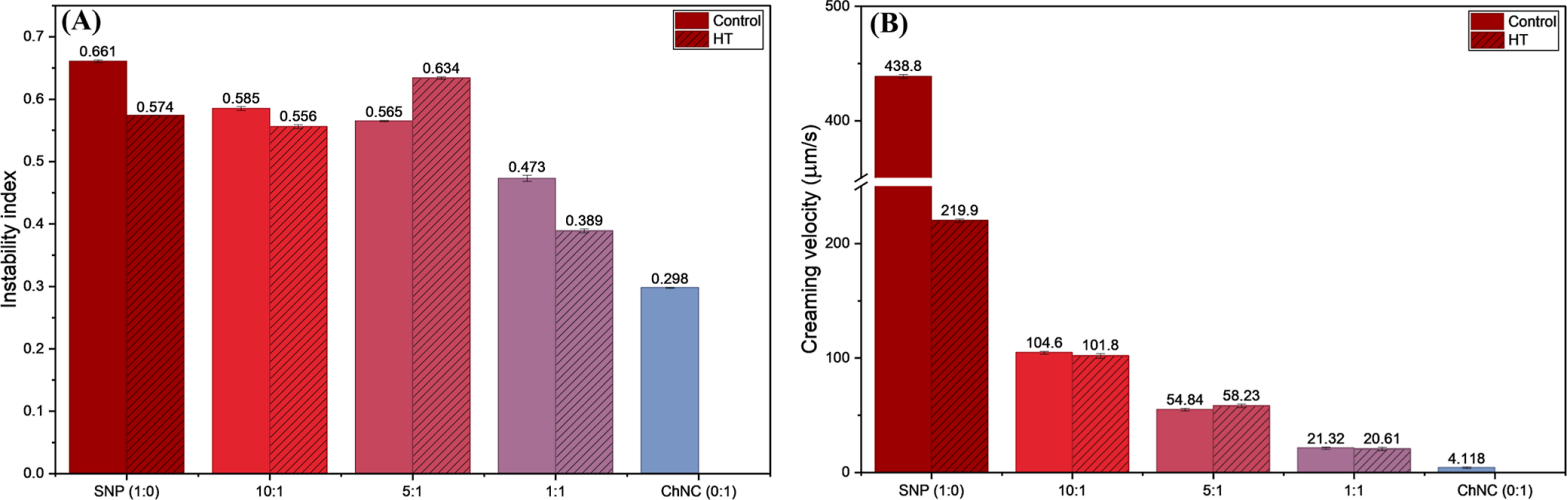
Instability index (A) and creaming velocity (B) obtained via accelerated stability analysis for the different SNP:ChNC ratio emulsions, both control and HT at 21 °C.

When looking at the creaming velocity (Figure 6B) of all of the samples, a more revealing result can be attained. There was an obvious decrease in the velocity with the addition of the chitin; even though the initial concentrations of chitin had an adverse effect on the stability as seen in Figure 6A, at the very beginning of the destabilization process, the chitin helps the system, evidenced by the low creaming velocity. Once again, the best results between the SNP and ChNC samples were displayed by the 1:1 sample.

The sedimentation velocity, which was evaluated for the aqueous phases (water and biobased particles with no oil), is defined as the rate at which dispersed particles settle to the bottom of the vial under centrifugation force and was lower for the SNP and chitin particles in the HT samples (Figure 7) than for the control samples, indicating enhanced suspension stability caused by the thermal treatment. The HT samples show an increase in sedimentation velocity with the addition of chitin, whereas the control samples do not show as much variation in sedimentation velocity with the increase of chitin fraction. This may be because the more flexible and softer gelatinized starch particles interact better with the ChNC to form denser particles congregates that sediment more quickly than when the two components are mixed but not preheated.^25,60^ The behavior observed in the sedimentation velocities of the samples mirrors the increasing viscosity of the aqueous phase with the addition of chitin nanocrystals (ChNC) (Table 1), highlighting the role of viscosity as a key emulsion stabilizing factor.

**Figure 7:**
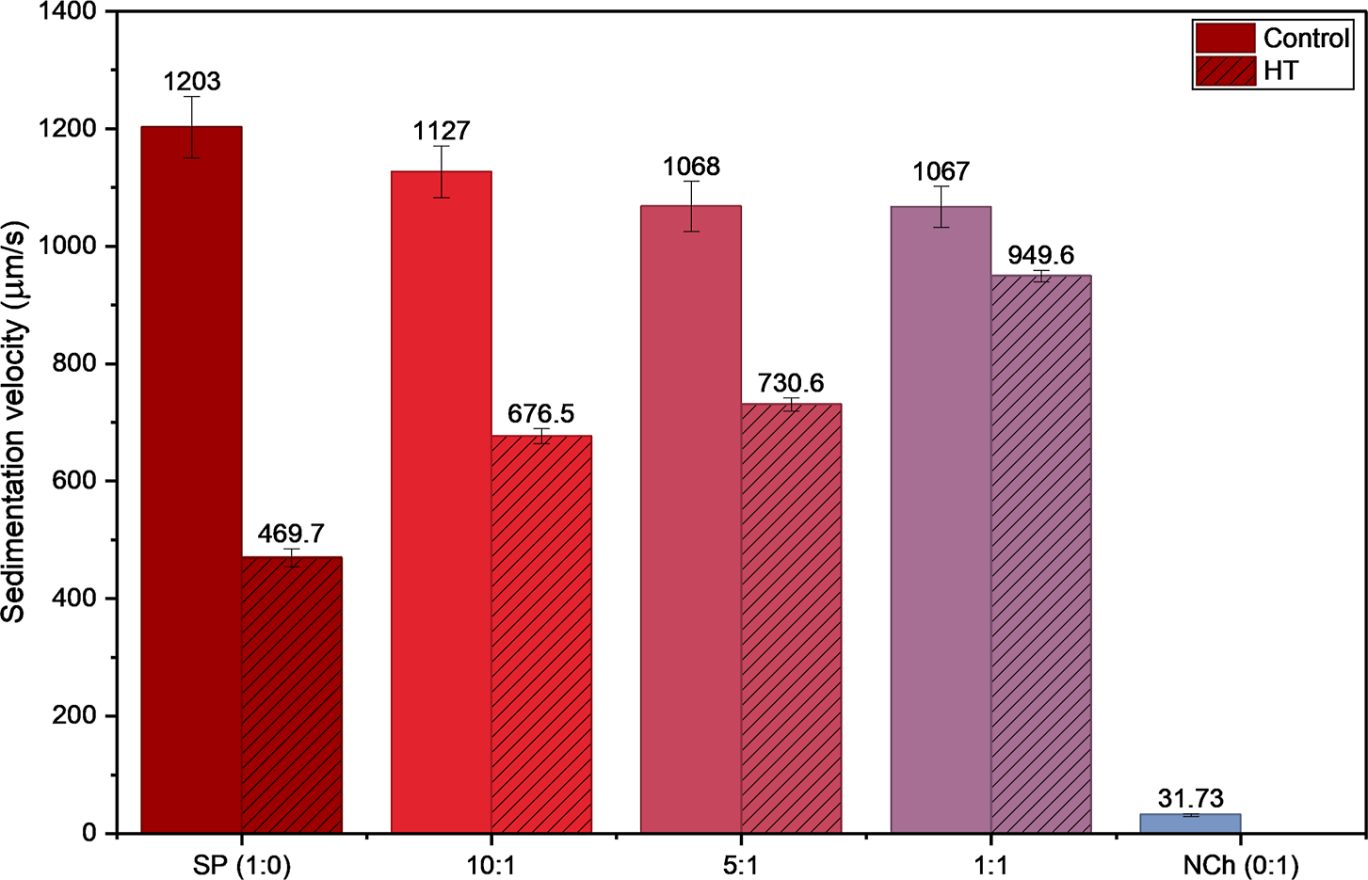
Sedimentation velocity obtained via accelerated stability analysis for the different SNP:ChNC ratio aqueous phase (with no oil), both control and HT.

### 3.7 Long-Term Stability of the Pickering Emulsions

The Pickering emulsions (SNP:ChNC ratios of 1:0, 10:1, 5:1, 1:1, and 0:1) were observed over 5 months regarding their stability and phase separation at 21 °C (Figure 8).

**Figure 8:**
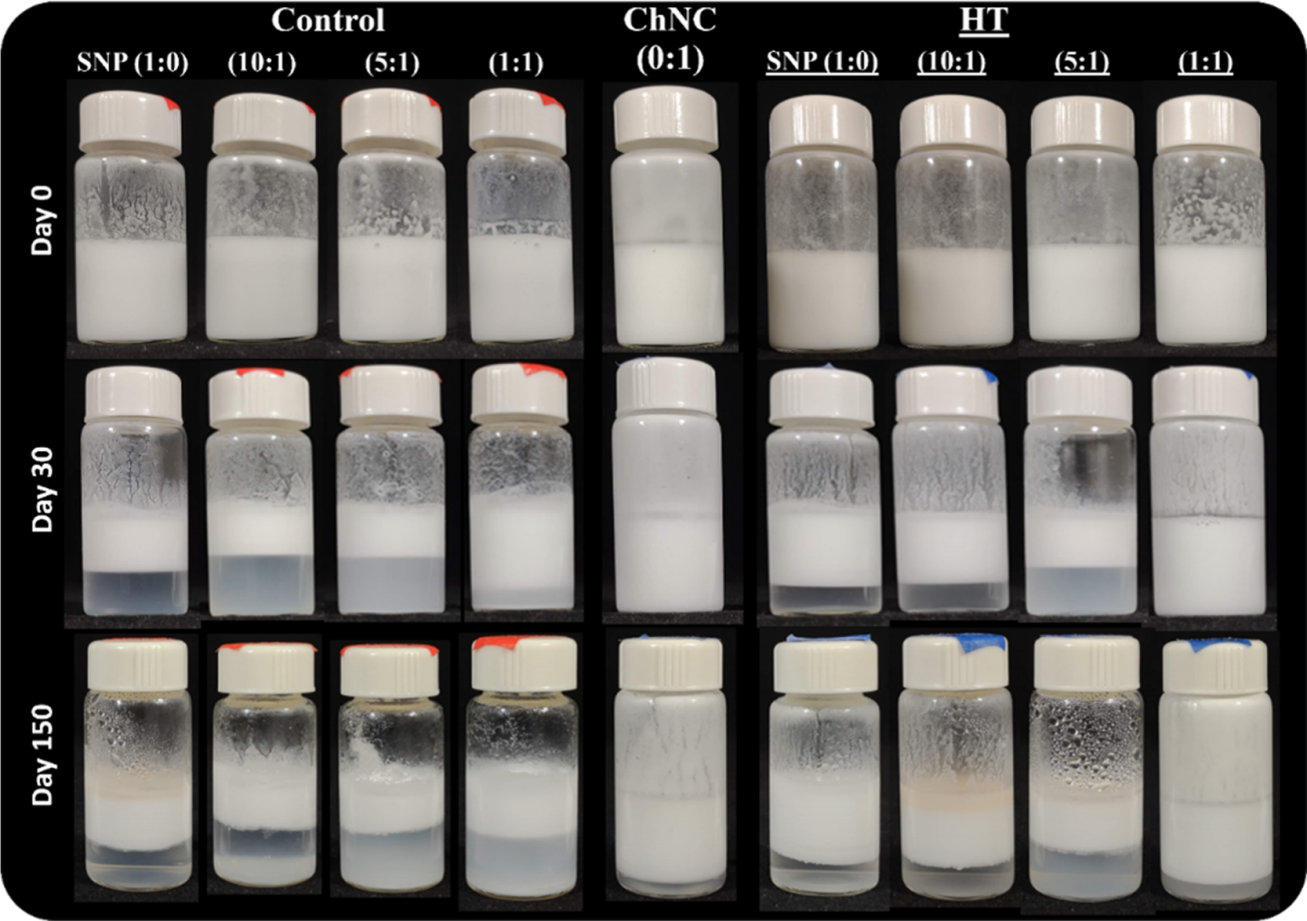
Visual appearance of the control emulsions (non-heat-treated) and the heat-treated (HT) emulsions with different ratios of SNP:ChNC over time (150 days) at 21 °C.

Pickering emulsions with differences in water/oil density and with large droplet sizes are prone to buoyancy effects, leading to creaming, which is the formation of a cream layer at the top and a serum phase at the bottom of the system.^61^ The visual appearance of the emulsions (Figure 8) indicates that their stability varied significantly depending on the proportion and treatment of the nanoparticles involved. Starch nanoparticles (SNP) without any thermal treatment proved to be unsuitable for long-term emulsion stabilization, as evidenced by significant creaming within 7 days after preparation (21 °C) (Figure 9B). Preheated starch moderately increases the viscosity of the aqueous phase of the emulsion (Table 1), which favors stabilization by hindering droplet mobility. However, high viscosity leads to a dense network, compromising the fluidity of the emulsion and droplet formation during homogenization.^62^ The initial addition of ChNC, particularly in samples 10:1 and 5:1, adversely impacted the stabilization process, leading to a higher creaming index compared with the SNP-only emulsion. However, when the ratio of ChNC was increased (1:1 sample), the two particles showed a synergistic effect, resulting in emulsions with the highest stability.

**Figure 9:**
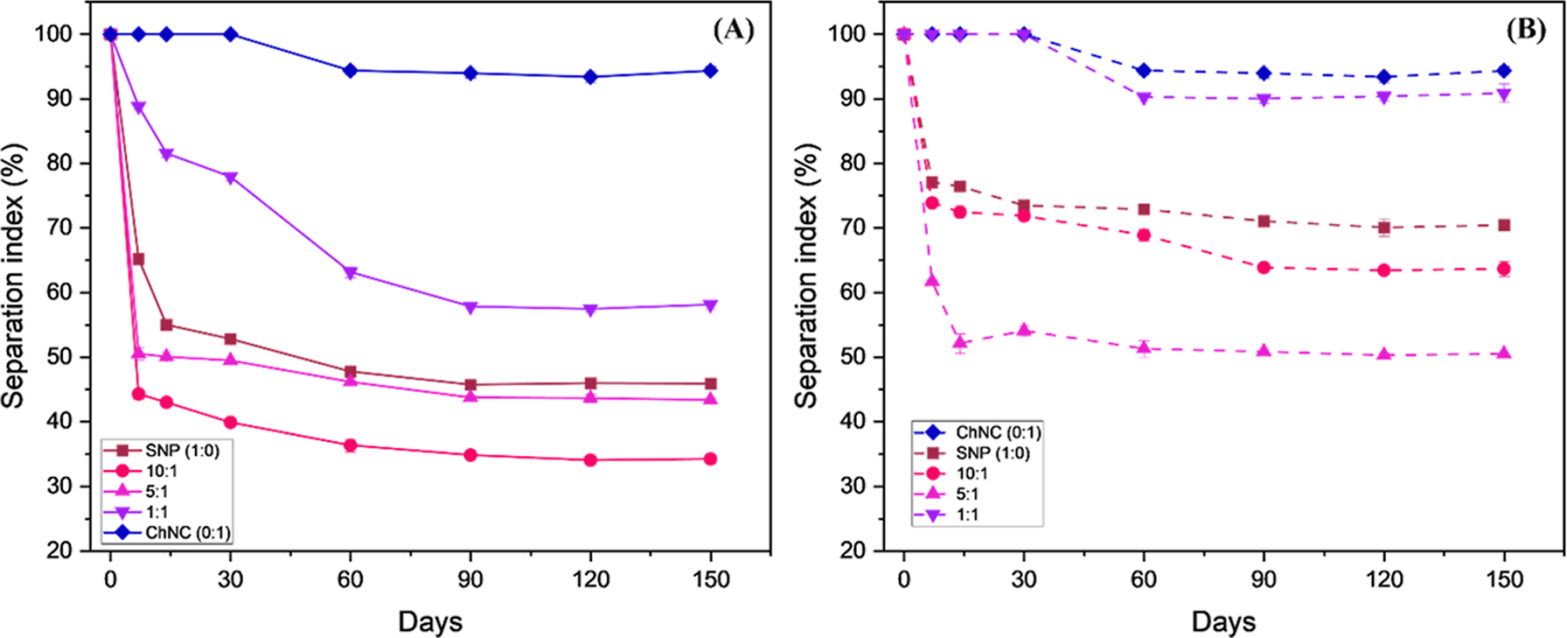
Separation indexes of the heat-treated emulsions control (A) and the HT emulsions (B) with different ratios of SNP:ChNC over storage time at 21 °C.

With the introduction of thermal treatment (HT samples), the samples exhibited a similar reduction in stabilization upon the initial addition of ChNC, mirroring the behavior observed in the control samples (Figure 9A). However, the 1:1 HT sample demonstrated results comparable to those of the ChNC-only emulsion, with creaming becoming apparent after only 1 month at room temperature. This can be attributed to the capacity of the smaller spherical gelatinized starch particles to be tightly packed in the oil–water interface with the chitin.^26^

After 5 months, the difference between the separation indexes of the 1:1 HT and the ChNC-only samples was <4% (Figure 9A). A better stability to creaming is found for the emulsions prepared by the combination of ChNC and SNP when compared to other starch-based systems, including pea-protein isolate^63^ and catechin,^29^ with the added benefit of the need for no chemical treatment to obtain the SNPs.

When compared with a chitin-only emulsion, the combination of SNP and ChNC offers several advantages, including reduced costs, improved digestibility, and enhanced potential for drug delivery. ChNC provides positive charges that enhance colloidal suspension stability and contributes to antibacterial and antifungal properties, which starch alone does not provide.^19,36,64,65^ Moreover, the two nanoparticles act under complementary stabilization mechanisms within the emulsion: SNP predominantly localizes at the droplet interfaces, while ChNC enhances interdroplet stability by forming a network that prevents coalescence, as confirmed by CLSM analyses.

### 3.8 pH and Temperature Stability

Figure 10 shows the creaming indexes for the studied HT emulsions under different temperature and pH storages on the day of preparation and then after 7 days of storage.

**Figure 10:**
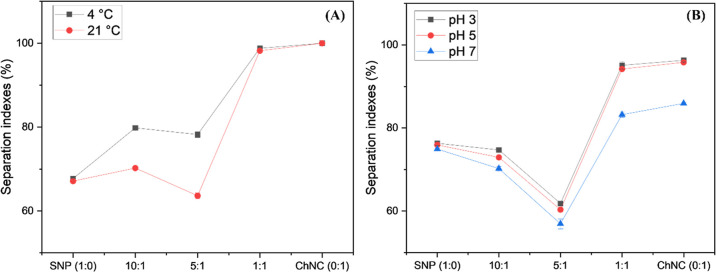
Separation indexes for the studied HT Pickering emulsions under different temperatures (A) and pH (B) conditions.

Figure 10A demonstrates that the stability of the emulsions improves when stored at cooler temperatures. This suggests that a potential final product utilizing this emulsion system could have an extended shelf life if kept in a cool environment such as a refrigerator. Enhanced stability is likely due to the thickening of liquids and colloidal systems, such as Pickering emulsions, at lower temperatures, which helps to inhibit coalescence.^66^

The emulsions also showed lower stabilities at higher pHs, and for those with higher ChNC content, this event is even more expressive, due to the better colloidal stability of chitin on acidic pHs, which would disrupt the effect of the ChNC on the bulk of the aqueous phase making it easier for coalescence to happen which leads to emulsion destabilization. But the emulsions still show satisfactory stability on the studied pHs which are the most common pH ranges for food products, indicating these emulsions would be stable to be used as ingredients in food products in this pH range.^67^

## 4 Conclusions

This study demonstrates that a sustainable particle system for stabilizing oil-in-water Pickering emulsions relevant to food applications can be created by combining starch nanoparticles (SNP) and chitin nanocrystals (ChNC). Mixed SNP/ChNC formulations do not necessarily outperform pure ChNC systems in all stability metrics but enable comparable stability while reducing the ChNC content, maintaining low viscosity, and improving formulation practicality.

A central finding is the strong effect of heat treatment applied to the aqueous phase prior to emulsification. Heat-treated SNP exhibits enhanced interfacial activity, leading to smaller droplet sizes and improved resistance to droplet growth over time. Among the mixed systems, the 1:1 SNP:ChNC heat-treated formulation showed the most balanced overall performance.

Microscopy and stability analyses indicate effective particle adsorption at the oil–water interface and restricted droplet mobility due to structuring of the continuous phase. In mixed systems, SNP mainly contributes to interfacial stabilization, while ChNC reinforces stability through the formation of a network in the aqueous phase. Beyond physical stability, the SNP/ChNC system offers advantages in terms of sustainability, cost reduction, and the potential to tailor the nutritional properties. Overall, these results highlight heat-treated SNP–ChNC combinations as a promising platform for designing food-grade Pickering emulsions.
